# Spectroscopic Methods Used in Implant Material Studies

**DOI:** 10.3390/molecules25030579

**Published:** 2020-01-29

**Authors:** Sławomir Lach, Przemysław Jurczak, Natalia Karska, Agnieszka Kubiś, Aneta Szymańska, Sylwia Rodziewicz-Motowidło

**Affiliations:** Department of Biomedical Chemistry, Faculty of Chemistry, University of Gdańsk, Wita Stwosza 63, 80-308 Gdańsk, Poland; przemyslaw.jurczak@ug.edu.pl (P.J.); natalia.karska@ug.edu.pl (N.K.); agnieszka.szczepanik@phdstud.ug.edu.pl (A.K.); aneta.szymanska@ug.edu.pl (A.S.)

**Keywords:** auger electron spectroscopy, X-ray photoelectron spectroscopy, Raman spectroscopy, photoluminescence piezospectroscopy, fluorescence microscopy, methods, implant, surface

## Abstract

It is recognized that interactions between most materials are governed by their surface properties and manifest themselves at the interface formed between them. To gain more insight into this thin layer, several methods have been deployed. Among them, spectroscopic methods have been thoroughly evaluated. Due to their exceptional sensitivity, data acquisition speed, and broad material tolerance they have been proven to be invaluable tools for surface analysis, used by scientists in many fields, for example, implant studies. Today, in modern medicine the use of implants is considered standard practice. The past two decades of constant development has established the importance of implants in dentistry, orthopedics, as well as extended their applications to other areas such as aesthetic medicine. Fundamental to the success of implants is the knowledge of the biological processes involved in interactions between an implant and its host tissue, which are directly connected to the type of implant material and its surface properties. This review aims to demonstrate the broad applications of spectroscopic methods in implant material studies, particularly discussing hard implants, surface composition studies, and surface–cell interactions.

## 1. Introduction

The enormous amount of scientific methods used today is a consequence of the multitude of hypotheses and problems tackled by the research community. One such problem is understanding the origin and complexity of interactions between materials. Extensive studies revolving around various materials and their interactions have led to the conclusion that in the majority of cases, the surface properties of the material have the most profound effect on its interplay with its surroundings and govern its short- and long-term behavior. Useful methods of study that focus on material interface should be able to deliver (i) qualitative information, (ii) quantitative information, (iii) be of a nondestructive character, (iv) tolerate a broad type of substrates, and (v) be relatively simple in use and interpretation. Of course, no method spanning all these conditions exists, and therefore scientists often choose several approaches to gather the necessary data. Nevertheless, there is a group of methods that can meet most of the requirements which fall into the general category of spectroscopic methods.

Over the last decades, spectroscopic methods have been applied in numerous studies revolving around analytical, biophysical and physical, or organic chemistry and material science. Examples of applied spectroscopic methods include agriculture and food analysis [1,2], environmental problem analysis [3,4], catalyst evaluation [5,6], chemical mapping [7], polymer analysis [8,9], biological surfaces [10], cellular [11,12,13] and tissue analysis [14,15,16,17], biosensing [18,19], and clinical applications [20,21,22].

Spectroscopy, according to the International Union of Pure and Applied Chemistry (IUPAC) [23], is defined as “the study of physical systems by the electromagnetic radiation with which they interact or that they produce. In certain types of optical spectroscopy, the radiation originates from an external source and is modified by the system, whereas in other types, the radiation originates within the system itself”. Following this definition comes a broad group of techniques, many of which, for example, X-ray photoelectron spectroscopy or Raman spectroscopy, are known for their vast scope, applicability, and reliability.

One of the areas that combines features of various scientific fields, for example, already mentioned material science and biophysical chemistry, and has benefited from developments in spectroscopy is implantology. Several past decades are marked with incredible advances in this area, especially regarding dental implants and bone prosthetics. Implant geometry, material type, and implant coating have all been the subjects of intensive studies, however, it has been proven that the material and coating type have the biggest impact on implantation success. Evaluation of material and coating characteristics have very often been performed with the use of suitable spectroscopic methods.

**Figure 1:**
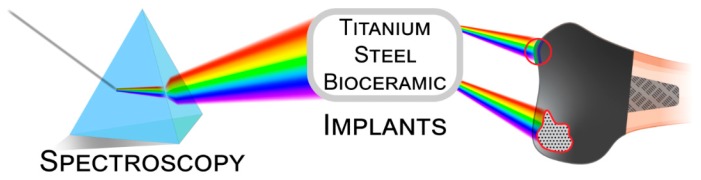
Spectroscopy utilizes parts of electromagnetic spectrum for analysis and studies of the processes occurring on the surface and in the subsurface regions of implants manufactured from metal alloys and bioceramic materials.

The purpose of this review is to bring together and highlight the most common methods used in the evaluation of implants and implant-tissue interfaces, with respect to the types of materials used. This review focuses on “hard” (metal and ceramic) implants and includes methods that focus on cell behavior analysis which is important for the analysis of the degree of material impact on the surrounding tissue (Figure 1).

## 2. Metal-Based Implants

Metal implants [24,25], especially titanium-based implants, have taken the major share of the implant market. Owing to the ease of manufacture and relatively low costs they are the number one choice for dental and orthopedic applications, with new advancements being constantly brought to light [25,26]. The broad use of metal-based implants made them a subject of extensive evaluation utilizing spectroscopic methods.

### 2.1. Steel, Titanium, and Titanium Alloy-Based Implants

#### 2.1.1. Auger Electron Spectroscopy

Auger electron spectroscopy (AES) is considered a very well-known analytical technique utilized in material science for surface-related studies. It uses an electron beam, which is directed at the analysis spot (ranging from 10 nm to 5 µm) and ejects electrons from the sample of interest, usually a conductive or semiconductive material. With the sampling depth varying between 1 to 5 nm (2 to 10 atomic layers), AES offers very high spatial resolution. Due to its nature, AES carries several limitations of which probably the most important ones are (i) the inability to analyze elements of fewer than three electrons (such as hydrogen and helium) and (ii) significantly better detection of lighter elements. Both of these restrictions stem from the physics behind the Auger process which is beyond the scope of this review and can be found elsewhere [27,28]. Finally, due to the utilization of an electron beam, AES can lead to sample deterioration or destruction, and therefore beam current densities need to be adjusted, especially when the sample is of great importance.

AES has many applications, mainly revolving around film growth [29,30,31], surface chemical composition [32,33], grain boundaries analysis [34,35], or quality control surface analysis in integrated circuits production [34,36]. Early reports of AES used in implant-related studies date back to 1985 to 1986, when Sundgren et al. [37,38] focused on understanding the mechanisms governing reactions occurring at the interface between a stainless-steel implant and tissue. Supported by scanning electron microscopy (SEM) and depth profiling, authors of the study presented several interesting results. First, the variation of the nature and thickness of the (mainly) chromium-based oxide present on the surface of the implant was attributed to the location of the implant. The thickness of an initially present 50 Å-thick layer varied with respect to the location of the implant; implants located in the cortical bone had the thickness of the oxide unchanged while the ones located in the bone marrow were thicker by a factor of three to four. Furthermore, in both cases, calcium and phosphorus were incorporated into the oxide. On the other hand, the oxide layer on implants located in the soft tissue within the oral cavity was only 1.5 times thicker as compared with the initial thickness of 50 Å. Interestingly, in the last case, instead of calcium and phosphorus, sulfur was present within the oxide layer. The authors proposed several hypotheses, along with possible mechanisms to justify such a discrepancy of the oxide thickness which included: (i) the difference in oxygen concentration along the implant, (ii) electron transfer mediated by proteins adsorbed on the surface, (iii) pH variation, and (iv) a combination of high protein concentration accompanied with high concentrations of the electrolyte.

The occurrence of calcium has been explained as a result of its co-precipitation together with phosphate ions, which can originate from various sources in body fluids. Because experimental results have pointed to the thickest oxide layer to be found in the bone marrow, a place of high metabolic activity requiring highly energetic phosphate bonds to be broken, it is reasonable to consider this as an additional driving force behind the oxide formation. Interestingly, it has been reported that the amount of sulfur also varied with respect to the placement of an implant, with its highest amount traced back to implants located in the oral part of the body. It has been unclear at that moment, what was the main sources of sulfur identified on the implant surface. Among many possibilities, authors have mentioned proteins adsorbed on the surface of the implant and chondroitin sulfate-containing proteoglycans, although none of these sources is location specific, therefore, it has not been possible to determine why only oral-region implants contained sulfur.

**Figure 2:**
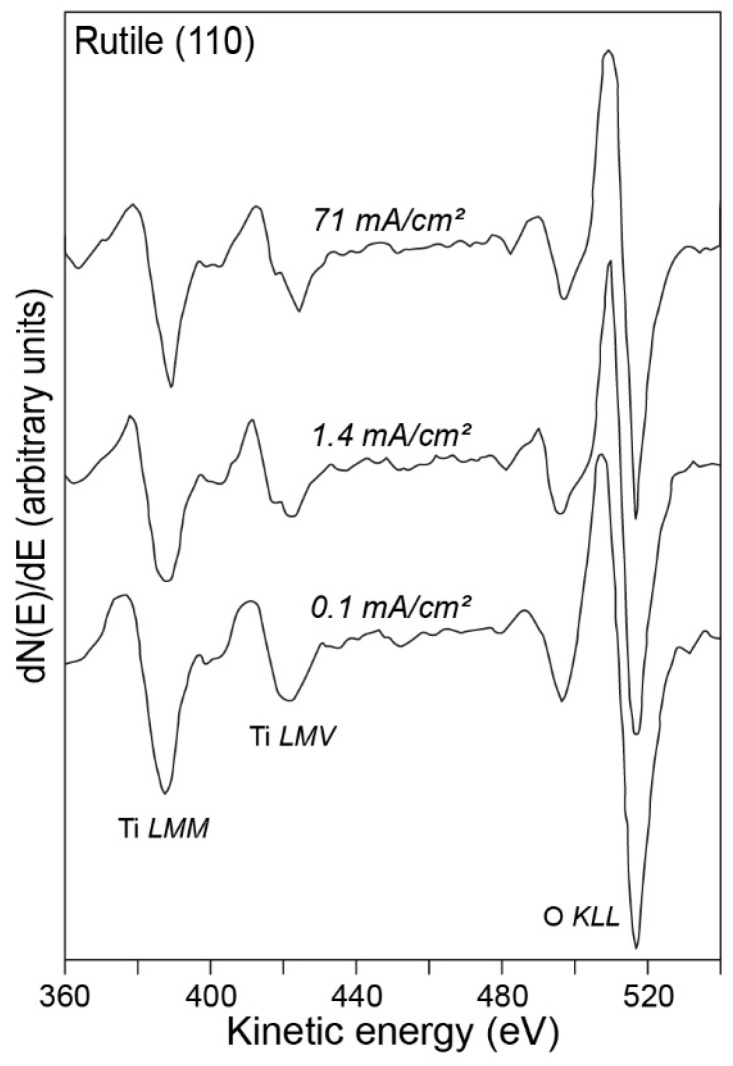
An Auger electron spectroscopy (AES) spectrum, for rutile TiO_2_ single crystal at different primary electron beam intensities. Figure adapted with permission from [39].

An early example of utilizing AES in an analysis of titanium implant surfaces [39] dates back to the late 1990s. The author of the referred paper focused on establishing a relationship between surface properties and the biological response to the material. In this example, titanium surfaces were prepared in the following four different ways: (i) machining, (ii) electropolishing, (iii) thermal oxidation, and (iv) nitrogen ion implantation. All these methods have been, among other techniques, also the subject of AES analysis. Furthermore, the author compared the results obtained for pure titanium with the results for the Ti6Al4V alloy and added observations resulting from biological studies. An AES spectrum from the above-cited paper is shown in Figure 2.

**Figure 3:**
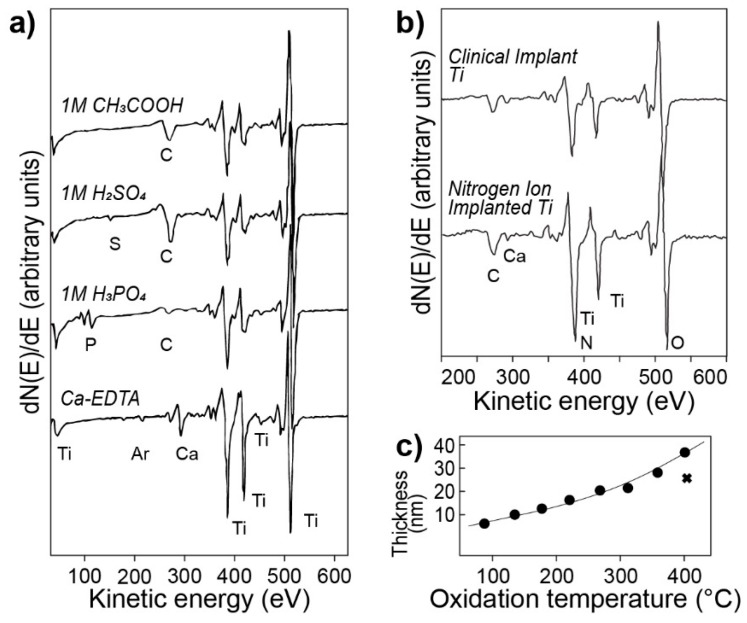
Examples of AES spectra recorded during various experiments. (**a**) Anodic oxides of titanium prepared in several electrolytes, (**b**) comparison between machined (top) and nitrogen ion implanted (bottom) samples, and (**c**) thickness of an oxide layer as a function of temperature. Figure adapted with permission from [39].

The data clearly demonstrate that the Auger peaks can be used to distinguish between different titanium oxides, while, at the same time, underlining the dependency of the results on analysis parameters (electron beam energy and dose). An example of such dependency can be observed in the change of the Ti LMV transition peak (≈420 eV) in Figure 2, starting from the bottom spectrum, where the electron beam intensity and dose are lowest and below the threshold for significant oxygen desorption to occur, and finishing with the topmost spectrum, with a probable Ti_2_O_3_-like oxide composition due to excessive beam intensity. These results have been utilized for comparison with data obtained with X-ray photoelectron spectroscopy (XPS), explained in detail later, and, as previously mentioned, for surfaces prepared in different ways. The AES results have been in agreement with the XPS data, which helps to establish the reliability of AES spectroscopy. Examples of further experiments are shown in Figure 3. Here, AES has been successfully employed to assess the composition of titanium oxides prepared in various electrolytes (Figure 3a), to compare clinical titanium with nitrogen ion-implanted titanium (Figure 3b), and to evaluate the thickness of the oxide layer as a function of temperature (Figure 3c).

To conclude, in the above referred publication, the author relied on AES as an important tool for implant surface characterization. Used side-by-side and compared with X-ray photoelectron spectroscopy, AES results have been verified and in agreement with XPS data. Despite its potential, in recent years AES has been gradually substituted with X-ray photoelectron spectroscopy. Nevertheless, the existence of such a method should be kept in mind, especially for depth profiling applications.

#### 2.1.2. X-ray Photoelectron Spectroscopy also known as Electron Spectroscopy for Chemical Analysis

X-ray photoelectron spectroscopy (XPS) is probably the most often utilized method for surface studies, often mentioned alongside Auger electron spectroscopy [40], with which XPS shares many similarities such as applicability to all elements, in addition to hydrogen and helium (due to the physics behind the process [41,42]), and possibility of sample deterioration. The main difference between the techniques is the utilization of an X-ray photon in the case of XPS in contrast to an electron beam used in AES. Importantly, this difference leads to greater sensitivity of AES as compared with XPS, as the Auger electrons are ejected from regions closer to other atoms as compared with core-level electrons ejected in XPS. Furthermore, XPS can be applied to a greater variety of materials, adding polymers, glasses, and other insulating materials to the group of conductors and semiconductors typically studied with AES. XPS is used everywhere where surface or thin film composition plays a critical role in performance, including nanomaterials [43,44], photovoltaics [45,46], catalysis [47,48], corrosion [49], and general support of industrial research and manufacturing [50].

**Figure 4:**
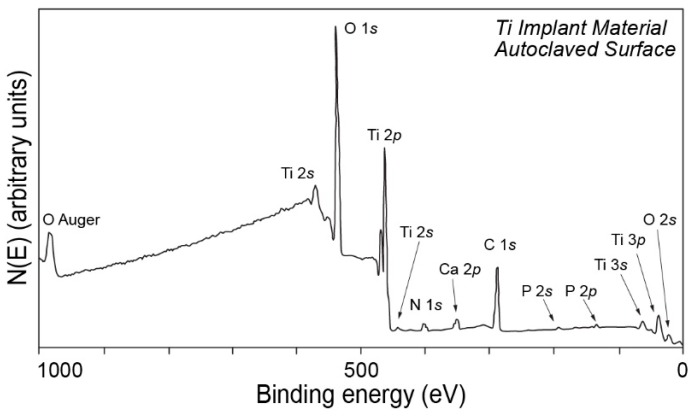
X-ray photoelectron spectroscopy (XPS) spectrum of a titanium implant surface revealing its composition. Figure adapted with permission from [39].

One of the early examples referring to the use of XPS in implant surface studies was presented by Lausmaa et al., where X-ray photoelectron spectroscopy was used in a so-called survey surface analysis to determine the initial composition of the surface of machined and autoclaved titanium surface and to trace the changes in the composition after performing further experiments (Figure 4).

**Figure 5:**
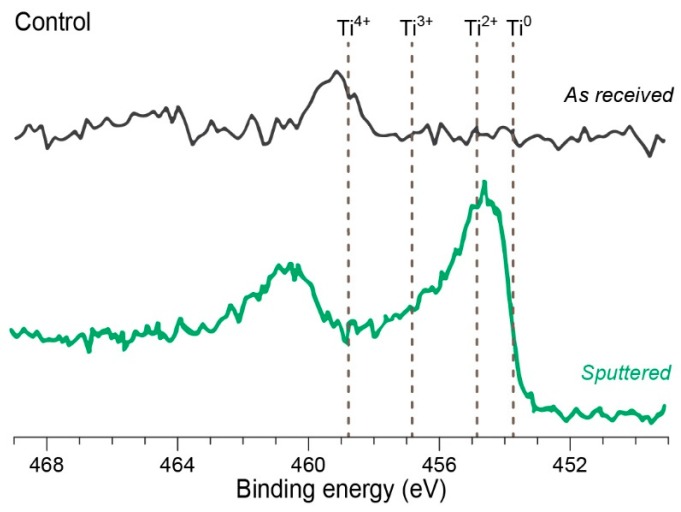
Examples of XPS spectra of titanium implant surfaces. Figure adapted from [51] with permission from The Royal Society of Chemistry.

In the next example, the authors studied the effect of bacteria on a dental implant surface, with the main focus on the morphology and corrosion of the implant, utilizing XPS [51]. In their experiments, the authors compared results from the “as received” implants with implant samples that had been sputtered in order to remove superficial layers and contaminants from the specimen surface, thus, revealing other oxidation states (Figure 5). The obtained results confirmed that the presence of *Streptococcus mutans* and *Porphyromonas gingivalis* in the implant environment can lead to the changes in the oxidation states of titanium and further corrosion of the metal surface.

**Figure 6:**
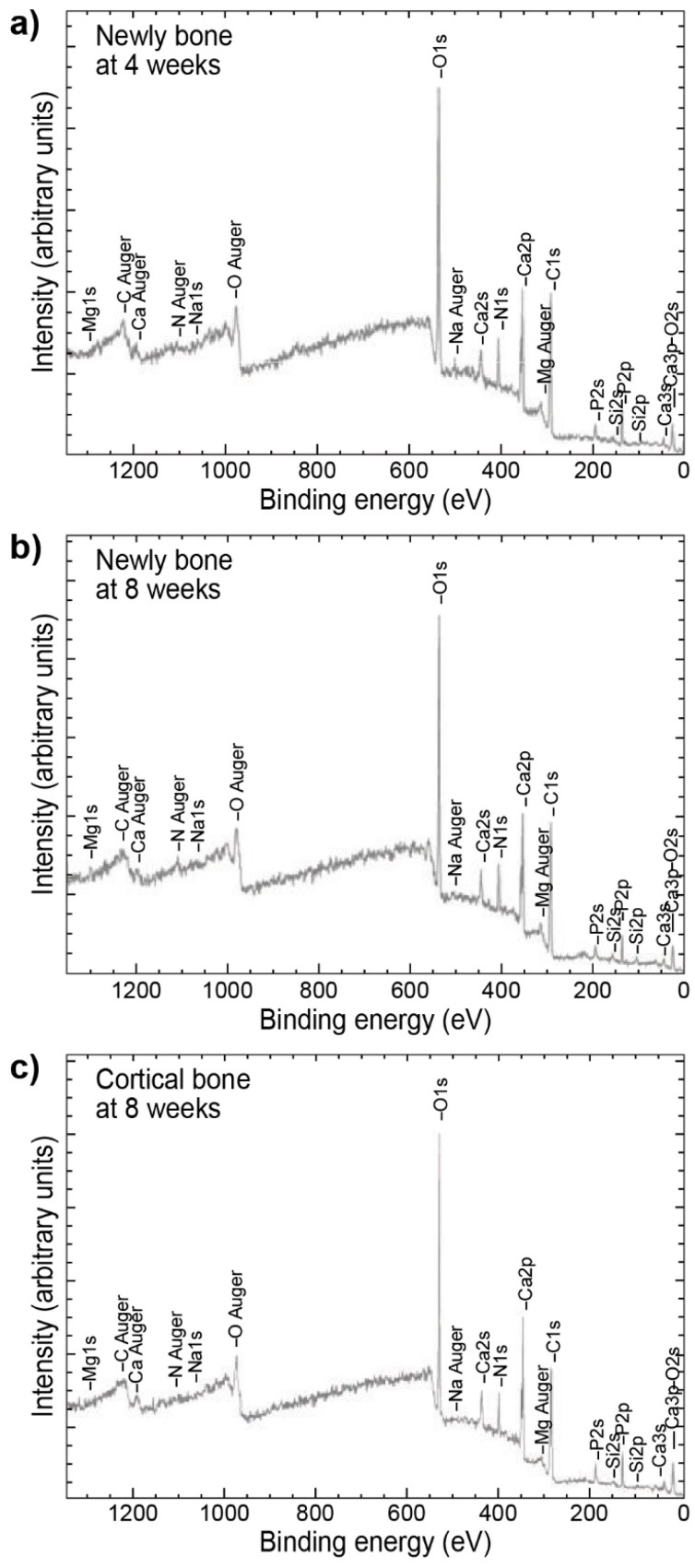
XPS spectra of newly formed bone after (**a**) four weeks, (**b**) eight weeks in the proximity of the titanium implant, and (**c**) cortical bone, as a control. Figure adapted with permission from [52].

The following example does not exactly focus on studies of implant surface, nevertheless it is worth mentioning, since it presents XPS as a tool capable of studying changes and differences in bone quality between newly formed bone, sampled from the vicinity of a titanium alloy implant. By comparing the data to that of cortical bone (acting as a control) in combination with sampling at different time points the changes were tracked within the newly formed bone [52] (Figure 6).

**Figure 7:**
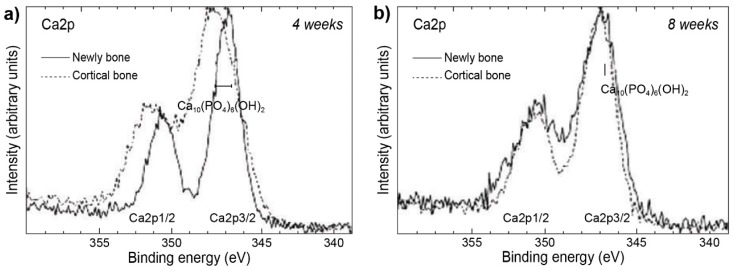
Narrow scan XPS spectra comparing newly formed and cortical bone after (**a**) four and (**b**) eight weeks. Figure adapted with permission from [52].

Although the spectra presented in Figure 6 appear to be almost identical, differences become apparent after taking a more detailed look at the shapes of some peaks and their relative intensities differing between spectra. Furthermore, comparison of the results taken at four and eight weeks for the newly formed bone, with the results for the cortical bone revealed more details and track the changes in the bone structure taking place during bone maturing (Figure 7).

The XPS data was also utilized for quantitative analysis of both, newly formed and cortical bone (Table 1). The authors concluded from the results that, after eight weeks, the newly formed bone has a strong physicochemical resemblance to the cortical bone due to maturation and bone metabolism.

X-ray photoelectron spectroscopy has become the “go to”, most widely used tool for surface analysis. Its applicability to a broad range of materials combined with the delivery of both, quantitative and chemical state information from the sample’s surface, are the pillars of its success and popularity among researchers.

#### 2.1.3. Raman Spectroscopy

Raman spectroscopy is being considered as a complementary technique to infrared spectroscopy (IR) due to its sensitivity to vibrational modes, which are either not observed in IR or yield only weak absorption bands. On the molecular level, Raman spectroscopy relies on the polarization of the electron cloud of the chemical bond dependent on the vibration of the bond-forming atoms. For details on the physics behind these phenomena, the reader is pointed towards more specialized texts [53,54]. Since molecular vibrations are unique for every molecule, Raman spectra are often viewed as a type of molecular “fingerprint”, widely utilized in semi-qualitative and semi-quantitative analysis in biology [55,56], chemistry [57,58,59,60], geology [61], life and material sciences [62,63], as well as in pharmaceutical [64], cosmetics [65], and semiconductor industry [66]. This wide spectrum of applicability of Raman spectroscopy is supported by its speed of data acquisition and lack of special needs for sample preparation.

**Figure 8:**
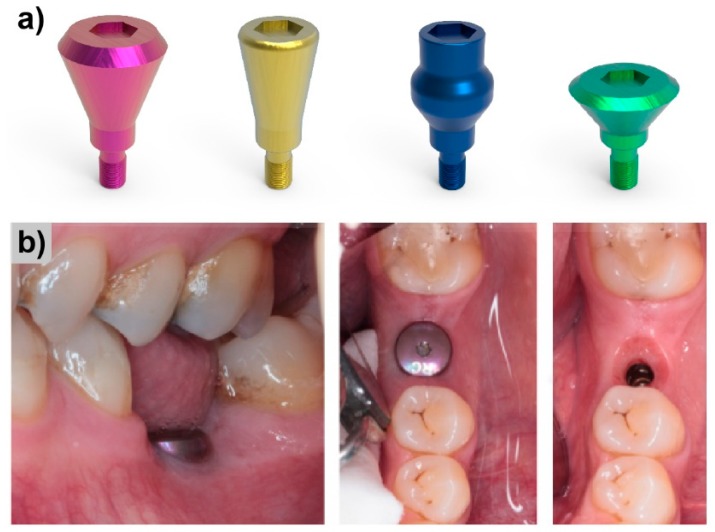
Implant abutments. Examples of (**a**) of various geometries of abutments and (**b**) their placement in the oral cavity. Pictures (**b**) reproduced with permission from *Reusing Titanium Healing Abutments*, Darin Dichter, 4 April 2016.

The field of implantology has benefited substantially from Raman spectroscopy. Considering the conditions to which an implant is exposed, it is a must to include surface studies to analyze implant surface reactions to the outside stimuli. An example of utilization of such surface studies are titanium implant abutments (Figure 8a) used in modern dentistry as a tool for soft tissue conditioning prior to proper implant placement (Figure 8b).

**Figure 9:**
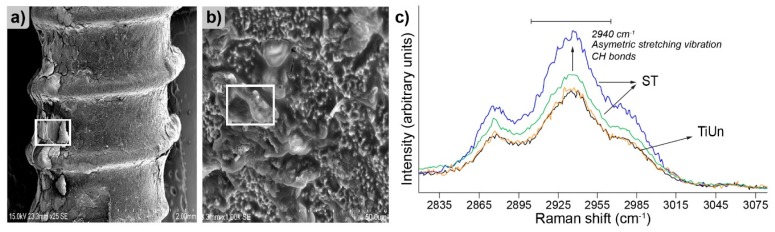
Scanning electron microscopy (SEM) images: (**a**) Representing the bone-like augments on the Straumann ST implants followed by (**b**) a higher magnification of the implant surface, the boxed regions have been subjected to Raman analysis and (**c**) Raman spectrum depicting organic matrix deposition on Straumann ST and TiUnite systems. Figure adapted with permission from [68].

Usually, an abutment is analyzed with several techniques in order to characterize any changes in surface crystallinity, morphology, and elemental composition, with an emphasis on the thickness of the oxide layer and both, the identity and orientation of the crystal phase [67]. A typical approach starts with surface scanning electron microscopy (SEM) analysis followed by Raman spectroscopy of regions of particular interest [68]. Such an example is illustrated in Figure 9, which depicts results from a study focused on surface alterations and possible bone formation on the surface of failed dental implants (either Straumann ST type or TiUnite), which were removed due to any given reason. First of the two SEM micrographs (Figure 9a), presents fragments of a fibrinous matrix with a less osseous surface, whereas the second (Figure 9b) shows a similar region with higher magnification. The presented surface reveals a significant degree of mineralization which can only take place due to significant interactions happening on the bone–implant interface. The presence of such features on the surface confirms a significant contact level between the implant and the bone.

Raman analysis was conducted to explore the origin of the augments, marked with the boxed regions in Figure 9b. One of the results of these experiments is presented in Figure 9c. Raman spectroscopy revealed a broad peak in the region specific for C–H bonds, in this case representing the bone organic matrix formed due to collagen deposition on the surface. Interestingly, the comparison between the Straumann ST and the TiUnite on the same spectrum revealed a higher degree of deposition in favor of the Straumann ST system.

Raman spectroscopy has been the method of choice for analysis of TiO_2_ phases present on root surfaces of commercial dental implants [69]. The presence of TiO_2_ on the metal surface is of tremendous importance for the chemical stability and durability of such an implant. However, an even greater impact can be attributed to the phase type of the oxide layer. Because interactions on the oxide–tissue interface play a crucial role in the long-term implant behavior, facilitating the support of the implant by the bone often happens due to hydroxyapatite growth on the metal surface, which can be affected by the type of the oxide phase. Hydroxyapatite does not readily grow on amorphous TiO_2_, contrary to rutile or anatase-rich TiO_2_ surfaces, both being better substrates for hydroxyapatite, yielding substantially better growth, which can have a significant influence on osseointegration [70]. As straightforward as it seems, obtaining a rutile or anatase-rich surface requires various treatments, for example, blasting, machining, plasma treatment, and anodization, which yield implants varying in TiO_2_ phase composition. Keeping in mind the phase-dependent hydroxyapatite growth preference, the authors of the study evaluated several commercially available implants, focusing on the presence of amorphous/rutile/anatase phases with respect to the surface treatment method [70] (Table 2).

**Figure 10:**
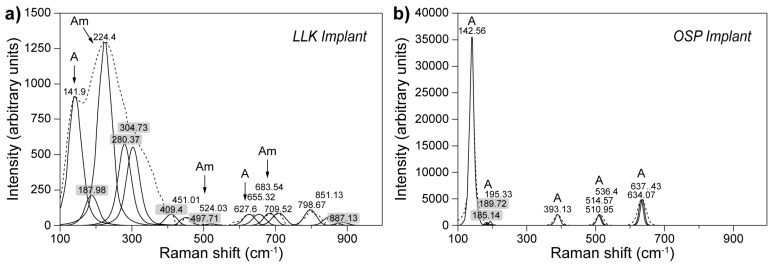
Raman spectra of (**a**) LLK and (**b**) OSP implant. Original spectra are denoted by a dotted line, curve fitting by a solid line. Figure adapted with permission from [69].

All the above-mentioned implants have been subjected to Raman analysis in order to evaluate the composition and TiO_2_ phase (or phases) present on the implant surface. Among many possibilities, the authors choose the intensity of the obtained signal as the first characteristics to divide the resulting spectra into three groups with maximum peak scale of: (i) < 800 a.u. for Group 1, (ii) 1250 up to 6000 a.u. for Group 2, and (iii) > 35,000 a.u. for Group 3. Within these groups, the authors analyzed the data and assigned signals with respect to reference materials. Exemplary spectra of implants yielding signals differing both in intensities and surface composition are presented in Figure 10. The tricalcium phosphate/hydroxyapatite blasted LLK implant (Figure 10a), assigned to Group 2, shows a broad peak profile, from which most of the signals have been recognized as belonging to amorphous and anatase TiO_2_ phases. On the contrary, the surface characteristics the OSP implant, assigned to Group 3, are a result of calcium-based anodization, and shows sharp and well resolved peaks, originating from anatase TiO_2_ phases.

Across other implants analyzed in the study, authors have shown that, besides already presented in Figure 10 anatase and amorphous phases, samples can contain Al_2_O_3_, brookite, or Ti_2_O_3_, in different proportions, depending on implant type or exclusively showing just one signal assigned to one of the phase types (the OSP implant in Figure 10b). Raman spectroscopy showed to be an outstanding tool for analysis of implants as received from the manufacturer and without prior preparations.

## 3. Bioceramic Materials

Bioceramic materials constitute a vast group of inorganic and nonmetallic compositions with numerous applications in medicine and medical devices [71,72,73], of which orthopedic and dental implant use covers the major share [74,75,76]. Furthermore, with ceramic materials gaining increasing attention and sparking a discussion of their versatility and superiority over titanium-based implants [77], it is important to note that not all ceramic materials are suitable for implant purposes. To address the issues of tissue attachment and post-transplantation material behavior has required countless modifications in the composition of such materials, ultimately leading to many examples, of which relatively few have achieved clinical success.

Considering the modus operandi, as well as surface and bulk interactions of ceramic-based implants, the analysis of crucial processes taking place in their vicinity has evolved over the years, followed by the development of bioceramics from simple bioinert materials through bioactive and resorbable materials, and finishing with bioceramics acting as scaffolds for cells [78]. In this section several examples of techniques used for the analysis of various ceramic materials are given, focusing on the most interesting examples from the groups of alumina and hydroxyapatite-containing materials.

### 3.1. Photoluminescence Piezospectroscopy

**Figure 11:**
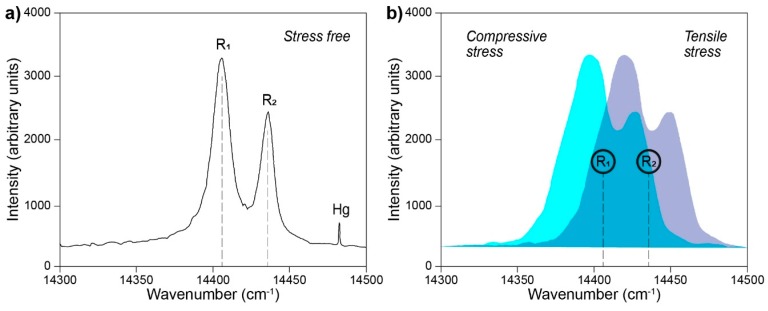
Photoluminescence piezospectroscopy spectra: (**a**) spectrum of polycrystalline Al_2_O_3_ resulting from the native presence of Cr^3+^ impurities and (**b**) hypothetical spectra of the material under compressive (cyan) and tensile (violet) stress with respect to the optical levels *R_1_* and *R_2_* (in circles) assigned from (**a**). Figure (**a**) adapted with permission from [85].

Photoluminescence piezospectroscopy (PLPS) is considered a technique of choice when examining surface imperfections, and nanoscale stress distribution on material surfaces are of significance. It is most frequently used for the analysis of alumina containing materials, typically ceramics [79,80], thermal coatings [81,82], or weld analysis [83]. The principle of this method is based on observations of the shifts of fluorescent Cr^3+^, as it is always present as an impurity (or added externally dopant) in aluminum, in response to applied stress. Utilizing a Raman optical probe, PLPS analysis can yield signals of intensity 10^6^ greater than obtained in typical Raman experiments, with the acquisition time of around one second. The obtained data is often compared to an unstrained reference sapphire sample. It is important to note that the peak position in the spectra is temperature dependent, and therefore temperature maintenance is of critical importance for reliable PLPS results [84]. An example of a typical PLPS spectrum is presented in Figure 11a, in which peaks *R_1_* and *R_2_* denote optical energy levels for Cr^3+^ [85]. Versatility of PLPS lies in its ability to distinguish between the type of stress being applied to the surface; compressive stresses shift the optical levels *R_1_* and *R_2_* towards lower wavenumbers (higher wavelengths), whereas tensile stresses shift them towards higher wavenumbers (lower wavelengths) (Figure 11b).

**Figure 12:**
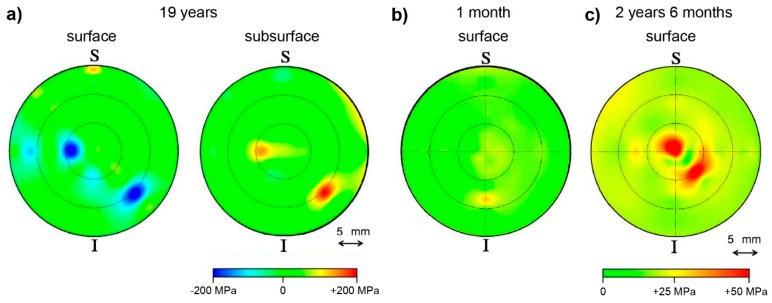
Stress patterns collected from implant femoral heads for (**a**) the sample exposed for the longest time in vivo and (**b**,**c**) samples exposed for short periods of time, one month and two years and six months, respectively. Letters S and I indicate the superior and the inferior locations of the joint during in vivo implantation. Figure adapted with permission from [85].

Data gathered during photoluminescence piezospectroscopy experiments have been used by Pezzotti et al. [85] to examine stresses on the surface and subsurface of hip joints exposed in vivo, starting from a period of one month, and finishing with nineteen years of exposure. Such a long time span allowed the authors to take a careful look at the performance of three generations of implants, all manufactured from Al_2_O_3_, differing in grain size of the material. The results of the analysis have been presented in a form of three-dimensional maps, examples of which are given in Figure 12.

Analysis of the data revealed intriguing patterns, especially in the case of the implant exposed for the longest time (Figure 12a). Both the surface and subsurface (30 µm below the surface) maps of the ball of the implant carried signs of stresses located in the same places, corresponding to the main wear zones, although surface stresses were shown to be compressive in nature, whereas the subsurface stresses were tensile. The authors explained this phenomenon by presenting a hypothesis of a local equilibrium being established between the surface and subsurface areas, which requires development of stress fields of opposite sign. Importantly, the authors point to a possibility that the presence of stress fields of such a magnitude could be the cause of surface degradation, along with grain detachment and formation of debris. A different trend was found on samples retrieved after a short-term implantation (Figure 12b,c). As expected, in both cases, the samples showed significantly lower stress fields, due to the relatively short term of usage. However, both cases differed in the type of stresses revealed on the surface, the stresses for the short-term used implants showed to be tensile in nature. Furthermore, the authors pointed out that the averaged stresses tend to evolve over time, starting from tensile for short-term used implants, through a point of stress annihilation, eventually leading to compressive values.

### 3.2. Raman Spectroscopy

Raman spectroscopy has already been introduced as a tool utilized in hard implant surface studies. In this section, its applications in hydroxyapatite-related studies are presented. Hydroxyapatite (HA) is one of the best, if not the best, well-known bioceramic materials to date. It owes its importance to its chemical similarity to bone itself, at the same time being biocompatible, bioactive, and stable. HA has been the subject of many outstanding reviews, focusing on its synthetic methods [86,87], applications in dentistry and oral implantology [88], biomedicine [89], and as an element of composite materials [90,91]. Typically, HA is used as an implant coating, to improve the fixation of the implants with bone tissue.

**Figure 13:**
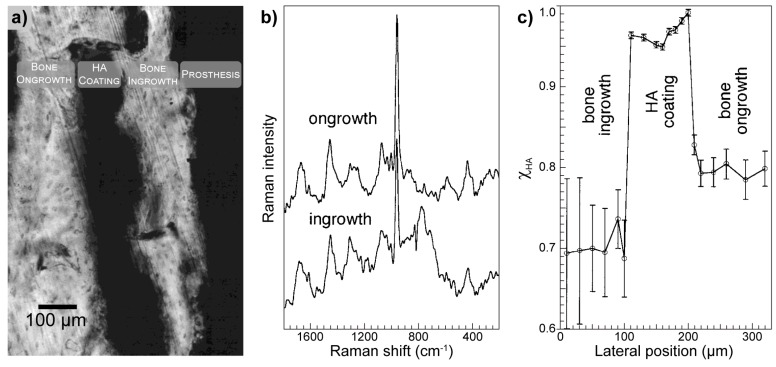
Canine specimen: (**a**) microscopic section of a coated implant, eighteen months after implantation. Grey parts of the image represent bone tissue, while the hydroxyapatite (HA) coating and the prosthesis are in black, (**b**) Raman spectra of the ongrowth and ingrowth part of the bone and (**c**) hydroxyapatite content (χ_HA_) at different lateral positions. Figure adapted with permission from [92].

Dippel et al. studied mineralization of bone tissue and HA coating of integrated hip and knee prostheses of human and canine specimens, particularly utilizing Raman spectroscopy to investigate the level of bone integration by analyzing the vicinity of the prosthesis or its coating and the bone–coating interface. Additionally, the authors investigated the spatial distribution of different phosphate structures [92]. One of the obtained results is presented in Figure 13. All samples were compared to appropriate controls in the form of uncoated implants to assess the effect of HA.

The microscope image of the canine specimen sample (Figure 13a) shows the HA coating detached from the implant after six months of full-weight bearing, creating space for the bone ingrowth to be formed. The authors compared the ongrowth and ingrowth parts of the bone to discover a 13% difference in HA contents in favor of the ongrowth part (Figure 13b,c). Additional observations have shown the distribution of HA to be at similar levels all the time, independent of the composition of the coating and history of the implant. The content of HA changed, beginning from a maximum in the direct vicinity of the implant, decreasing to a minimum towards the boundary of the coating to increase again from the side of ongrowth bone.

Simmler et al. performed a similar study of HA coatings, although in this case, the authors turned their attention towards the integration of HA-coated implants in order to establish the timespan of full-bone mineralization [93]. For this purpose, Raman spectra was acquired in the form of lateral scans from the surface of the implant, reaching in the subsurface area of the bone tissue, to determine the amount of protein and inorganic components. The obtained results differentiated between HA form present in mature bone tissue and synthetic HA present in the implant coating. Furthermore, it was shown that implants residing in the body over a period of eighteen months contained the same amount of HA across the bone-implant interface as compared with the mature bone tissue. At the same time, the content of synthetic HA was reduced. On the contrary, after six months the samples did not achieve full mineralization and the content of synthetic HA was substantial, with the synthetic form found at positions beyond the implant coating, showing the active involvement of the coating in the mineralization of new bone tissue.

**Figure 14:**
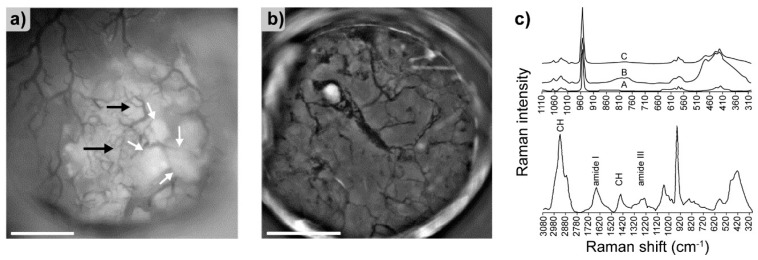
(**a**) Images from intravital observations of HA implanted within the calvaria, six months after surgery. Black arrows point to blood vessels within the bone and white arrows to implanted material. Scale bars correspond to 1 mm. (**b**) Eight months after surgery, the implanted β-TCP is not present. (**c**) Raman spectra of HA powder prior implantation (A), intravital hydroxyapatite spectra in rabbit calvaria after three (B), and twelve weeks (C). After implantation (top) and intravital bone two weeks after implantation (bottom). Figure adapted with permission from [94].

One of the most challenging studies utilizing Raman spectroscopy was performed by Penel et al. [94]. Employing special titanium bone chambers, the authors utilized intravital Raman spectroscopy to monitor bone composition directly in living animals, focusing on HA and β-tricalcium phosphate (β-TCP). The chambers were implanted in the femur and the humerus of New Zealand female rabbits, and therefore spectral and optical observations could be made without any additional surgery (Figure 14a,b).

Spectra of HA taken at different times are presented in the top panel of Figure 14c. During the study, no significant frequency shifts were detected. The authors performed additional experiments in order to analyze the composition of the bone tissue two weeks post implantation (Figure 14c, bottom). Data revealed several modes characteristic for carbonate apatite typically encountered in bone tissue (${\mathrm{PO}}_{4}^{3-}$: 1073, 1035, 1048, 960, 604, 587, 450, and 430 cm^−1^; ${\mathrm{CO}}_{3}^{2-}$: 1103 and 1073 cm^−1^), together with signals attributed to the presence of collagen (amide III: 1200 to 1300 cm^−1^, amide I: 1600 to 1700 cm^−1^, and CH groups: 2800 to 3100 cm^−1^). Additionally, the eight month in situ observation allowed the authors to follow the integration of biomaterials with the bone tissue and revealed faster incorporation of β-TCP contrary to HA.

**Figure 15:**
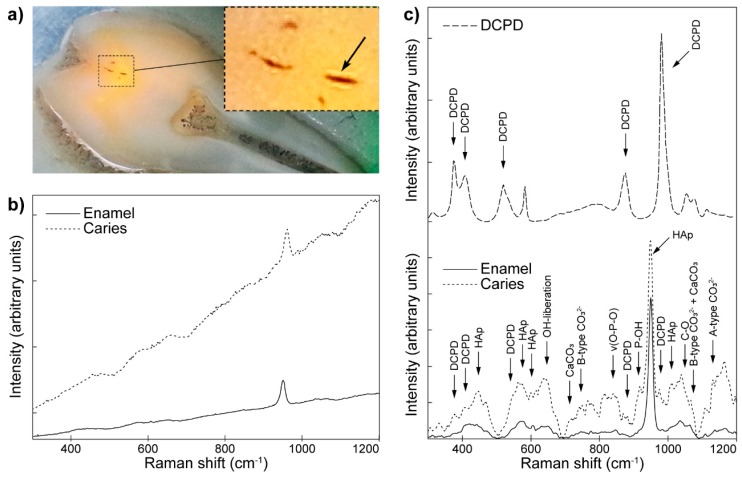
Analysis of tooth caries formation. (**a**) Example of a tooth sample irradiated by a green laser. The inset presents the irradiated part magnified, denoting with an arrow a light region of demineralization. (**b**) Raman spectra comparing enamel and caries form the region denoted by the arrow in inset of (**a**). (**c**) Raman spectra of dicalcium phosphate dihydrate (DCPD) (top) and overlaid enamel and caries (bottom). Figure adapted with permission from [95].

Quite an interesting example, though not exactly regarding bioceramic implants, shows the utilization of Raman spectroscopy along with fluorescence microspectroscopy to study phase transformations in solid tissues of human teeth occurring during tooth caries formation, delivered by Seredin et al. [95]. First, the authors investigated the teeth areas with enamel carious fissure (Figure 15a). Fluorescence experiments performed on the sample revealed fissures, visualized as dark spots with surrounding lighter areas (magnified in the inset of Figure 15a, marked with the arrow). The reason for the observed increase of fluorescence around the fissures is explained as a result of the decrease of crystallinity and absence of HA in preferable orientation, marking irreversible changes taking place in the organo-mineral complex along with the removal of the mineral components.

Raman spectroscopy has been chosen as the perfect tool to analyze the micro-sized fissures (marked with the black arrow in the inset of Figure 15a). The results of the analysis are presented in Figure 15b, confirming the increased intensity of caries region as compared with the enamel. The authors took further efforts to extract details revealing differences in the surface composition caused by the phase transformations. More detailed Raman experiments (Figure 15c) shed light on the differences between the two regions of interest. The top spectrum in Figure 15c presents dicalcium phosphate dihydrate (DCPD, ${\mathrm{CaHPO}}_{4}\cdot 2H_{2}O$), proposed as an intermediate in apatite mineralization and dissolution processes. Its signals have been recognized on the carious fissure spectrum, overlaid with the enamel signals at the bottom of Figure 15c. Additionally, the data shows significant variations in signal intensity in the fissure spectrum combined with an increase in intensity of signals near 775 cm^−1^ and 1075 cm^−1^, relating to ${\mathrm{CO}}_{3}^{2-}$, due to the substitution of the phosphate HA group in the structure by carbonate ions. On the enamel spectrum, signals corresponding to vibrational modes of ${\mathrm{PO}}_{4}^{3-}$, OH and ${\mathrm{CO}}_{3}^{2-}$ functional groups, characteristic for enamel’s inorganic component, are present. These data helped the authors shed some light on the mechanisms of fissure formation and the part played by hydroxyapatite in these processes. Their work further emphasized the importance of Raman spectroscopy and its complementarity with fluorescence microspectroscopy.

### 3.3. Diffuse Reflectance Infrared Fourier Transform Spectroscopy

**Figure 16:**
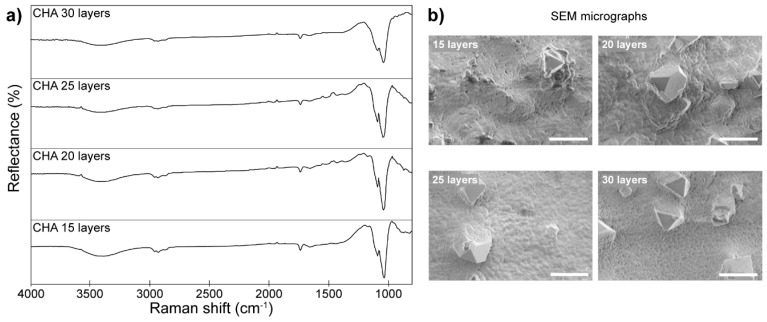
Sol-gel method derived calcium hydroxyapatite (CHA). (**a**) Diffuse reflectance infrared Fourier transform spectroscopy (DRIFTS) spectra of respective CHA layers accompanied by (**b**) SEM micrographs taken at magnification of 15,000×. Scale bars correspond to 3 µm. Figure adapted with permission from [102].

Diffuse reflectance infrared Fourier transform spectroscopy (DRIFTS) is a variant of IR spectroscopy, which takes advantage of the subsurface diffused part of the electromagnetic radiation, especially important if samples are yielding very little or no specular reflection, i.e., their surfaces are dull, rough, and lack shininess. The ability to analyze non-transparent specimens made of highly absorbing materials is the key advantage of DRIFTS. Other frequently mentioned advantages include: effortless sample preparation, very good signal-to-noise ratio, and ability to perform in situ measurements and quantitative analysis of surface species [96]. DRIFTS is most often applied to provide precious insights about mechanisms and reaction pathways [97,98,99], organic paints, and dyes [100,101]. It has also proven useful in implant-related studies. The following examples focused on analysis of hydroxyapatite coatings on stainless steel [102] and titanium implants [103]. In the first case, Jonauske et al. evaluated calcium hydroxyapatite (${\mathrm{Ca}}_{10}{\left({\mathrm{PO}}_{4}\right)}_{6}{\left(\mathrm{OH}\right)}_{2}$, CHA) coatings obtained by a sol-gel method as a means of protecting steel surfaces to aid their poor bioactivity and associated formation of fibrous tissue, issues typical for this material. In their experiments, a steel plate, initially roughened through physical treatment, was subjected to cycles (up to thirty) of spin coating of the CHA sol-gel followed by further annealing of the sample at 850 °C for five hours in air. So prepared, the samples were immersed into simulated body fluid (SBF) for two, three, and four weeks to evaluate the in vivo bioactivity of the coatings. Several methods were utilized to draw the necessary conclusions from the performed experiments, among them, DRIFTS was used to evaluate the formation and change in the composition following successful formation of CHA coating layers (Figure 16).

Analysis of the obtained spectra revealed almost no differences across the samples. Absorption bands in the range 1100–950 cm^−1^, typical CHA, are clearly visible, along with bands located at 1035 and 1090 cm^−1^, resulting from symmetric stretching vibrations in ${\mathrm{PO}}_{4}^{3-}$, and a broad band in the 3600–3300 cm^−1^ range attributed to the presence of water. An additional weak and broad band in the range 1550–1370 cm^−1^, attributed to ${\mathrm{CO}}_{3}^{2-}$ bending modes in CHA, was observed in all of the spectra. These results, accompanied by additional methods, confirmed the formation of good quality CHA coatings on the rough stainless-steel surface.

In the second case, Rigo et al. focused on titanium implants. However, the authors utilized a different strategy for covering the metal surface with HA [103]. HA was deposited by immersion of the implant in SBF along with bioactive glass. The glass released Ca^2+^, Na^+^, and K^+^ ions via an exchange with H_3_O^+^ present in SBF along with release of silicate ions, which adsorbed on the substrate surface and, eventually, led to formation of silanol (Si-OH) groups. These together with Ti-OH acted as nucleation sites for apatite growth. Nucleation was additionally stimulated by the earlier released Ca^2+^, Na^+^, and K^+^ ions, along with ${\mathrm{PO}}_{4}^{3-}$ ions present in SBF. Once the nuclei were formed, HA growth was spontaneous. So prepared, implants were inserted into rabbit’s tibia for eight weeks and afterwards analyzed accordingly. DRIFTS analysis was utilized in the early stage of the experiments, to assess and confirm the formation of HA.

## 4. Applications of Fluorescence Techniques for the Analysis of Implants and Implant-Surrounding Tissues

Biocompatibility and cytotoxicity are crucial aspects to be considered during the processes of design and optimization of properties of a new implantable material. These properties were studied in vitro and in vivo regarding the influence of the material on the behavior of prokaryotic and eukaryotic cells (e.g., proliferation, viability, and differentiation), which are, in most cases, chosen for the studies depending on the sight of implantation [104,105,106,107,108]. The most common methods to visualize the behavior of cells located in close proximity to the implant involve histopathologic staining [109,110,111] and fluorescence labeling [108,112,113]. This section of the review focuses on the latter one.

Since fluorescent staining is a very powerful tool for sensitive and selective labeling of a molecule of interest, principles of exemplary fluorescence microscopy techniques and different applications of the fluorescent visualization of cell–implant interactions or drug release are highlighted in this part of this review. Possible applications of the techniques including: (i) characterization and analysis of cells in implant-surrounding tissues, (ii) observations of cell behavior on the implant interface, (iii) evaluation of antibacterial properties of the implant surface and its cytocompatibility, and (iv) assessment of drug release will also be discussed (Table 3).

Contrary to the previously described spectroscopy techniques, the applications of fluorescent microscopy techniques do not vary as significantly when applied to different implant materials. The principles of fluorescent microscopy methods relay on the type of visualized cells and tissues and the fluorescent staining procedures. Therefore, contrary to the previous section of this paper, where the division with respect to material types was applied, here, we focus on the group of metal implants involving titanium and its alloys, nickel, platinum, cobalt, and chromium alloys. Additionally, two examples of active agent release from non-metal implants into the surrounding tissues are described in Section 4.2.5. Imaging Systems.

### 4.1. Fluorescence Labeling Methods

For monitoring of processes taking place at the cellular and molecular levels, fluorescence microscopy requires the sample to be labeled. Even though in some molecules the concentration of fluorophore is high enough for the autofluorescence to be used for visualization, the intrinsic fluorescence is in most cases weak, nonspecific, and of limited use [127]. There are several ways to induce fluorescence in a sample. The most common ways involve labeling with fluorescent dyes or combining with fluorescent proteins [128,129]. It should be stressed that fluorescence labeling exhibits some significant limitations, i.e., fluorescent dyes have limited stability, are susceptible to photobleaching, and can induce phototoxic effects [127].

**Figure 17:**
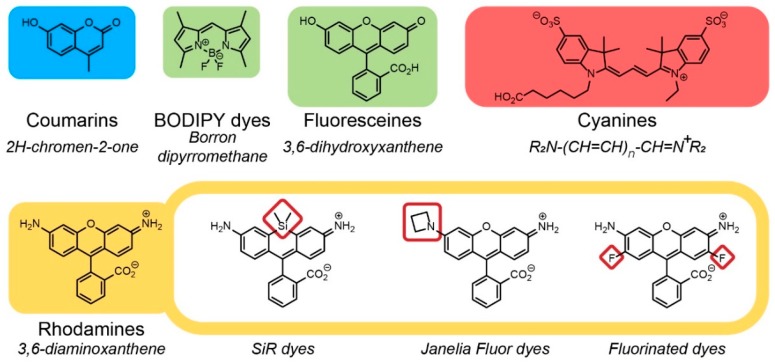
Examples of fluorescent dies with the background colored according to the wavelength maxima of fluorescence emission and examples of modifications of the rhodamines dye family (marked by the red square).

The simplest molecules used as fluorescent labels involve small organic compounds and fluorescent nanoparticles (NPs). On the basis of the core structure, organic fluorophores can be divided into such families as fluoresceines (e.g., calcein and FITC), coumarins, or rhodamines (Figure 17) [127]. All of them are prone to chemical modifications affecting their properties (e.g., wavelength range, brightness, and photostability), which is their key advantage over other fluorescent molecules [130,131,132]. Fluorescent NPs are often prepared from CdSe and ZnSe [133,134,135]. Their emission wavelength depends on the size and the material they are made of. A range of fluorescent dyes is also used due to their selective binding to a specific group of molecules. The examples of such involve 4’,6’-diamidino-2-phenylindole (DAPI) [136] and bisbenzimide trihydrochloride [116] which bind DNA thus being used for staining of cell nuclei, phalloidin, [137] acting as a fluorophore carrier and selectively binding F-actin, used for cellular filament staining, or tetracycline hydrochloride [138] which is readily absorbed into the bone. To monitor the antibacterial properties of surfaces, a live/dead bacterial viability kit is most often used [139]. It contains two nucleic acid stains that penetrate bacterial cells to a different extent. Green stain (e.g., SYTO^®^9 in the Thermo Fisher kit) penetrates the wall of all cells, whereas red stain (e.g., propidium iodide in the Thermo Fisher kit) only penetrates the damaged membrane of dead cells [139].

Immunofluorescence is a more advanced technique of molecule labeling. It relies on a highly specific binding of a primary antibody to a target molecule [140]. In some cases, the primary antibody can act as a fluorophore carrier, otherwise, a secondary fluorophore-containing antibody is introduced to the system to verify if the primary antibody bound successfully to the antigen-containing molecules.

The progress in the understanding of genetics has resulted in an increased range of possibilities to modify the processes of protein expression and utilize them to our advantage. As a result, fluorescent proteins became a popular direction for the labeling of biological samples. They can be expressed in vivo in genetically modified cells, for example, to monitor cell migration or co-expressed with other proteins in a form of a label to monitor the localization of the molecule [141,142]. Contrary to commonly used organic dyes, fluorescent proteins are most often nontoxic and allow studies of living cells.

### 4.2. Fluorescence Microscopy Techniques

#### 4.2.1. Fluorescence Microscopy

Fluorescence is one of the most popular contrasting techniques in microscopy. It involves the use of fluorophores or fluorochromes which absorb light at a specific wavelength and, after excitation, emit fluorescence [127]. Since the emitted fluorescence is much weaker (typically several thousand to million times) than the excitation light, a set of advanced filters is applied to selectively absorb the excitation light, and therefore only the emitted fluorescence to reach the detector [143]. This way, the observed structures are superimposed with high contrast against the dark background. The levels of fluorescence emitted by a sample are generally low, and therefore the sources of excitation light need to emit a light with high intensity [143]. The most often used light sources involve high-energy short-arc discharge lamps such as mercury or xenon burners. The more advanced fluorescence microscopes (e.g., confocal microscopes) use lasers as the light sources. The greatest advantage of the fluorescence microscopy is the presence of the fluorophore [127]. Although in some cases fluorescent microscopes do not work at high enough resolution to register the structure of specific molecules, the emitted fluorescence monitors spreading and localization of molecules within the sample.

Life sciences have found numerous applications for fluorescence microscopy. Focusing on implantology and new implantable materials only, they range from visualizing the bone metabolism to monitoring antibacterial properties of the new materials, facilitating, for example, the evaluation of different implantation techniques.

**Figure 18:**
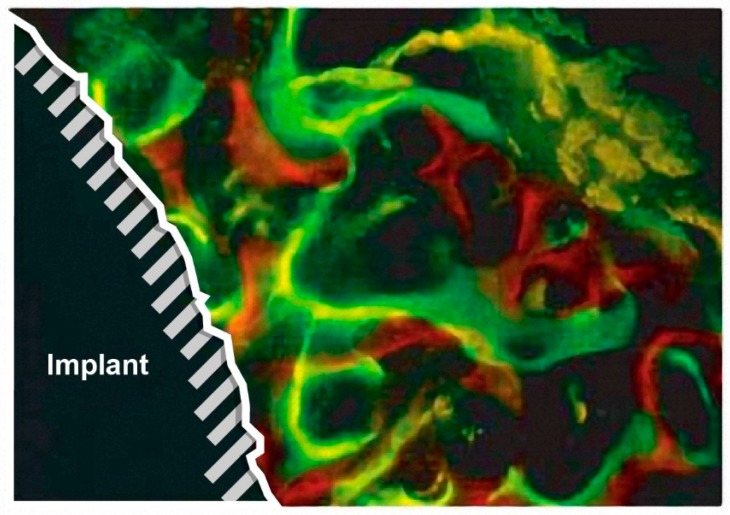
Fluorescence microscopy image of the bone compressed by the implant interface. Labeling with oxytetracycline (green/yellow) and alizarin complexion (red). Registered after two weeks of healing. Blue filter, ×40 magnification. Figure adapted with permission from [144].

One of them, the osteotome technique, aims at increasing the primary stability of dental implants in the posterior maxilla [114]. Although this method is well described and used in clinical routine, the experimental data on the post-implantation osseointegration are scarce. Therefore, in vivo polychromatic fluorescence labeling was applied by Nkene et al., to evaluate the osseointegration and morphology of newly formed bone tissue around the inserted implant [144]. Fluorescence labeling was used to follow the direction and the topographic localization of new bone formation. For this purpose, four dyes (oxytetracycline, alizarin complexion, calcein, and xylenol) were supplied via intramuscular injection. After the bone formation, samples were isolated and analyzed. The obtained data showed weakly stained bone for the control group. The dyes accumulated around the implants in the sequence of their administration. A different pattern was shown for the osteotome technique group, where a strong signal in the whole compressed area was observed already two weeks after implantation (Figure 18). After healing periods of four and eight weeks, an even more pronounced dye apposition was observed in the compressed area and close to the implant surface. The study showed that the osteotome technique significantly increases osseointegration of implants.

The new implantation materials are designed to meet the highest standards of biocompatibility. To improve their properties surface modifications are often used. The studies on titanium implant surface modifications are among the most popular. As an example, one of the studies focused on the osteogenic and pro-angiogenic effects of a coating based on zinc (Zn) and magnesium (Mg) ions [115]. The ions were selected based on their high importance for cellular metabolism [145] and processes leading to adhesion of osteoblasts to orthopedic implant surface [146] and co-implanted into the titanium surface via plasma immersion ion implantation. During the study, the in vitro adhesion activity and viability of rat bone marrow mesenchymal stem cells (rBMSCs) on Zn/Mg surface was visualized by staining the cytoskeleton and cell nuclei with rhodamine-phalloidin and DAPI, respectively. The in vitro osteogenic induction and the pro-angiogenic effects of the ions were visualized by immunofluorescence labeling of osteocalcin (OCN) and HIF-1α (respectively) in rBMSCs and HUVEC cells. For this purpose, after the exposure to the corresponding antibodies (anti-OCN and anti-HIF-1α) the cells were stained with fluorochrome-conjugated secondary antibody and DAPI. The results of the study showed increased adhesion, proliferation, and differentiation of rBMSCs. The activation of HIF-1α (essential regulatory factor of VEGF) in HUVECs was also increased, thus, enhancing angiogenesis. Additionally, due to the antibacterial properties of the studied coating, a certain inhibitory effect towards *Porphyromonas gingivalis, Fusobacterium nucleatum,* and *Streptococcus mutans* was observed.

Bioceramics represent another sort of implantable material compared to previously mentioned titanium. One of the studies describing applications of orthopedic bioceramics aimed at evaluating the properties and cytocompatibility of silicon carbide (SiC) ceramics, synthesized from different substrates obtained from wood specimens [116]. The studied SiC was coated with a hydroxyapatite layer to increase its osseoinductive properties. To verify the osseoinductive properties of the hydroxyapatite coating and SiC surface, the NIH 3T3 fibroblast cell line was used. The cells were stained with bisbenzimide trihydrochloride and phalloidin to detect cell nuclei and F-actin, respectively. The fluorescence microscopy showed that the coating increased the implant cytocompatibility.

Multicomponent coatings are an advanced strategy which affect several properties of an implant at the same time. An example of such coating exploited the synergetic antibacterial properties of ZnO nanorods and silver NPs (AgNPs) [117]. Additionally, poly(lactic-*co*-glycolic acid) (PLGA), a biodegradable and highly biocompatible material, was used as one of the coating components to increase surface stability and control the release of the active agents over time. It was assumed that the PLGA/Ag/ZnO composite coating of titanium implant should release dual antibacterial agents (zinc and silver) to improve surface antibacterial efficiency and enhance biocompatibility (PLGA). To evaluate the properties of the multicomponent coating, its influence on the morphology of osteoblastic cells (MC3T3-E1) and viability of bacteria (*Staphylococcus aureus* and *Escherichia coli*) was monitored. To visualize alive and dead bacteria and assess the antibacterial properties of the coating, propidium iodide and acridine orange were applied. The MC3T3-E1 cells were stained with FITC-phalloidin and DAPI to monitor their morphology. The data obtained from the study showed that, after optimization of active agent concentrations, the coating provided a long-lasting antibacterial activity and good cytocompatibility.

Fluorescence microscopy is in a state of constant, rapid development, with new advancements in probes and equipment appearing almost every day. As a result, new possibilities become available, for example, to increase imaging resolution or to perform advanced three-dimensional (3D) imaging. The need for specialized techniques adjusted for specific samples has resulted in the development of a range of state-of-the-art fluorescence techniques including X-ray fluorescence microscopy, confocal microscopy, or total internal reflection fluorescence microscopy.

#### 4.2.2. X-ray Fluorescence Microscopy

X-ray fluorescence (XRF) microscopy is used for quantitative measurements and imaging of trace elements distribution in various kinds of samples [147]. In fluorescence X-ray microscopy the sample is irradiated with micro-focused and monochromatic X-rays [148]. The fluorescence spectra are obtained by changing the wavelength of the incident X-ray while scanning the sample point-by-point. The XRF microscopy shows what types and amounts of elements are contained in each point by detecting the emission of characteristic fluorescence X-rays from each specific element in the sample [149].

The advantages of X-ray fluorescence microscopy involve high sensitivity, multi-elemental detection capability, nondestructive analysis, minimum sample preparation, and the ability to analyze solid, liquid, or gas samples at atmospheric pressure [150]. The use of synchrotron X-ray fluorescence microscopy permits reduction in the spatial resolution up to the micron level, as well as significant improvements in the elemental sensitivity. The main limitation of this technique is strictly associated with its principle. X-ray fluorescence detects atoms depending on the specific wavelength emitted from the sample, but due to strong matrix effects, interferences can occur, causing weakening or strengthening of the primary X-rays, thus, questioning the accuracy of direct X-ray fluorescence analysis [151]. The applications of X-ray microscopy involve monitoring protein transportation, investigations of wet biology specimens, or elemental mapping of biological tissues [147].

Considering the fact that the nature and distribution of metal accumulated within the surrounding tissue helps to evaluate the level of corrosion of electrode implants, Spiers et al. applied XRF to verify the amount of the material released from the implant into the surrounding fibrous tissue [118]. The study aimed at analyzing the implant-surrounding tissue, after long-term implantations (implantations in 1978, 1983, and 1998; material retrieved post-mortem in 2007). The use of X-ray fluorescence microscopy allowed for the detection and differentiation of various forms of accumulated platinum, for example, platinum NPs which were released most probably as a result of corrosion, and platinum ions which later formed complexes with proteins most probably released as a result of electrolytic processes.

In another study, micro X-ray fluorescence (µXRF) microscopy (a method with a spatial resolution with a diameter many orders of magnitude smaller than conventional XRF) was used to monitor the release of cobalt and chromium from a Co-Cr alloy-based implant into the surrounding bone tissue [119]. The study aimed at determining if it is possible to prevent metal release by covering the implant surface with a thin layer of titanium dioxide utilizing the atomic layer deposition method. The µXRF mapping determined that the titanium oxide layer significantly decreased the amount of metal released into the tissue. Additionally, the increase of the layer thickness decreased the release even more. However, the release was not entirely averted. The TiO_2_ layer also prevented the corrosion process of the alloy in physiological conditions.

A similar study was performed by Uo et al. who applied X-ray scanning analytical microscopy (XSAM) to monitor the release of nickel from nickel implants [120]. The data form XSAM mapping was compared with results from histological analysis and showed that the area of the nickel spreading obtained from XSAM mapping was highly consistent with the area around the implant exhibiting inflammation, detected during histological analysis.

#### 4.2.3. Confocal Microscopy

Confocal microscopy is commonly applied for visualizing specific objects within living and fixed cells or tissues [152]. High resolution of the technique registers detailed structures [153]. Contrary to the standard fluorescence microscope, confocal microscope is built so that the laser light irradiates only a defined spot at a specific depth in a sample, the rest of the sample left out of focus. When samples are scanned in one plane in a raster pattern, a two-dimensional (2D) view of one sample plane is formed. Scanning an object one plane after another creates a 3D model. Advanced confocal microscopes containing different filters can irradiate the sample with several lasers during one measurement for simultaneous analysis of samples stained with several dyes at once [154].

The main advantages of confocal microscopy involve the possibility of registering images with high resolution and contrast [155], combined with the ability to prepare 3D models of the image data gathered during the experiments. The possibility of in vivo measurements up to c.a. 200 µm in depth is another benefit of this technique. As a result, it is possible to, for example, monitor the entire epidermis and superficial layer of the dermis. Considering the technique’s limitations, the depth of imaging is limited by the optical penetration and signal-to-noise ratio. It should also be stressed that, similar to the other fluorescence techniques, fluorescence confocal microscopy struggles with photobleaching and cytotoxicity of the fluorescent probes.

**Figure 19:**
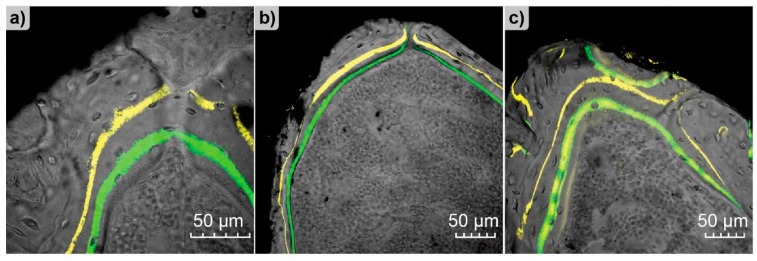
Laser scanning confocal microscopy images of bone remodeling after different types of implantation procedures: (**a**) sham surgery, (**b**) ovariectomy, and (**c**) strontium coated implant insertion. Labeling performed with tetracycline hydrochloride and calcein. Registered 4 weeks post-implantation, x400 magnification. Figure adapted with permission from [112].

The confocal microscopy can monitor changes in bone morphology. As a consequence, it is feasible to visualize the effect of an implant on bones in predisease or diseased states. One of such states is osteopenia, a preclinical state of osteoporosis [156], which causes the bone weakening and makes the use of orthodontic implant anchorage and tooth implants difficult or even unadvised. Therefore, Liang et al. performed a study with the aim to elaborate an improved method for increasing the success rate of dental implantation, avoiding at the same time drug therapy [112]. For this purpose, titanium implants were coated with strontium, since it promotes bone formation. Fluorescence labeling was successfully used to visualize the processes of the new bone formation around the implant. The bone was stained with tetracycline hydrochloride and calcein after different periods. The canalization of the dye in the newly formed bone and distances between the dye lines showed the increased rate of mineralization and osseointegration (Figure 19).

Advances in modern medical engineering introduce different implant surface modifications [157] to enhance their biocompatibility. Collagen type I is one of the active agents used as a surface coating for orthopedic and dental titanium implants [104,107,108]. Ao et al. described the influence of such a coating on the in vitro behavior of human mesenchymal stem cells (hMSC) and in vivo new bone formation [108]. They utilized staining with phalloidin (actin filaments) and counterstaining with DAPI (nuclei) during in vitro tests to visualize the changes in cell morphology when in contact with the implant surface. For the purpose of the study, cell viability and spreading were visualized with the use of a live/dead bacterial viability kit (ab115347). In the in vivo study, the new bone forming around the implants was fluorescently labeled with calcein.

It often happens that different properties of the coating are studied in combination with the monitoring of cytocompatibility and antibacterial properties at the same time. Considering this strategy, Zhao et al. evaluated the properties of a nitinol surface functionalized with two-component coating, based on graphene oxide and gelatin molecules [121]. They verified cytocompatibility by measuring adhesion, viability, proliferation, and differentiation of MC3T3-E1 osteoblastic cells on the coated surface, while antibacterial properties of the surface were assessed by the viability of the *Escherichia coli* cells. For the purpose of visualization, the MC3T3-E1 cells were, first, the subject of an immunoassay with goat anti-mouse IgG-FITC followed by rhodamine-phalloidin and DAPI treatments to visualize the actin and cell nuclei, respectively, whereas the *Escherichia coli* cells were stained with live/dead bacterial viability kit (L13152, Molecular Probes).

#### 4.2.4. Total Internal Reflection Fluorescence Microscopy (TIRF)

The lateral and axial resolutions of conventional fluorescence microscopy are limited to c.a. 200 nm and 600 nm, respectively [158]. This prevents observation of some important biological structures and dynamics at the nanoscale. Advances in the development of microscopy apparatus have overcome this limitation by introducing super-resolution microscopy which comprises such techniques as stimulated emission depletion (STED), single-molecule localization microscopy (SMLM), or total internal reflection fluorescence microscopy (TIRF) [158,159,160].

TIRF is an example of super-resolution microscopy which finds its application in life sciences, where it can be used, for example, in detailed studies on the integration of biomaterials with the cells located in the surrounding tissues [127,159]. The main principle of TIRF takes advantage of the fact that most biological samples are placed on a thin glass coverslip, to exploit the properties of the interface [161]. During the experiment the coverslip is illuminated at a very low angle, limiting the excitation of the fluorophores only to those located in close proximity to the surface. In this way, the out-of-focus background noises, which are the central issue of microscopy in general, can be reduced and the axial resolution of the method can be increased even up to c.a. 100 nm [162].

Among many advantages of TIRF, the most prominent one is its excellent signal-to-noise ratio [163]. It is also worth mentioning that TIRF can be used to study processes and structures in live cells and increase cell viability in long-lasting experiments. This method exhibits low phototoxic stress due to low penetration of the sample by the excitation beam [164]. TIRF also resolves the problem of photobleaching. Since only a fraction of the available fluorophore is illuminated during the experiment and the constant flux from cytosol to the irradiated region provide new fluorophore molecules, the signal-to-noise ratio remains constant for a long period of time [165]. The main limitation of TIRF is the necessity to use a sample that is highly adherent to the microscope glass surface, because it has to be very close to the interface to be irradiated by the excitation beam [164].

The applications of TIRF in the field of biomaterials and implantology revolve mainly around description and explanation of processes occurring on the bone–implant interface such as the dynamics of cell surface adhesion [122]. Current data shows that the cell surface adhesion depends strictly on the properties of the surface [166] and the number of possible spots of cell–surface interactions (e.g., peptides, proteins) [167]. Since it is the ligands present on the surface of an implant that mostly determines its surface properties, a study was performed to determine the strength of attachment of bovine aortic endothelial cells to immobilized linear RGD peptides and fibronectin [122,123]. The data showed higher affinity of the cells to fibronectin. Another study focused on the dynamics of cell-implant contact [122,123]. Covering vascular grafts with endothelial cells is a promising direction for reducing the probability of post-implantation thrombosis. However, inserting the adhered cells into the environment with constant blood flow requires tight adhesion of the cells. It should also be considered that the flow can increase tension and compression applied to the cells causing constant stress to the cytoskeleton. In the study, TIRF was applied to monitor short-term response of the cells to the applied fluid forces. The study results indicated that the forces were not strong enough to influence the shape of the cell meaning that the cell did not move on the implant.

#### 4.2.5. Imaging Systems

The “top shelf” of imaging systems belongs to advanced, multifunctional apparatuses capable of acquiring fluorescence and bioluminescence data in vitro and in vivo, both in 2D and 3D modes (with varying options depending on the model). In the context of implantology these systems detect fluorescent material embedded in organic tissue in vivo in living organisms, and thus are applied, for example, in studies on drug release [124,126]. Xenogen IVIS-200 (PerkinElmer) or eXplore Optix (Advanced Research Technologies) are examples of systems used for fluorescence imaging in small animals. Simplified substitutes of commercial platforms exhibiting a narrower range of technical capabilities are also successfully utilized. What is important, they can be constructed and configured with regard to the exact experiment they are meant for [126].

**Figure 20:**
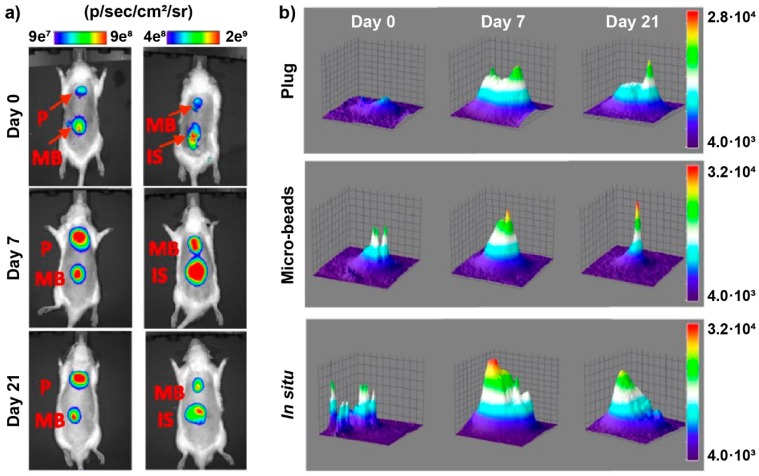
In vivo fluorescence analysis of hydrogels, with Xenogen IVIS-200: (**a**) whole-animal images of the implanted plugs (P), microbeads (MB), and in situ polymerized (IS) hydrogels and (**b**) representative 3D plots of the fluorescence signal distribution and intensity. Data registered 0, 7, and 21 days post-implantation. Figure adapted with permission from [124].

Multifunctional platforms for the release of active agents are the focus of modern implant engineering. One of the possible materials for such platforms are hydrogels, which can be formed into different shapes and combined with different active agents. An example of a hydrogel drug delivery platform was documented by Berdichevski et al. [124], who studied the integration of biodegradable hydrogel implants (rat model) containing vascular endothelial growth factor (VEGF). The study compared the release parameters and angiogenic properties of VEGF from three geometric configurations of the hydrogel, i.e., cylindrical plugs, microbeads, and in situ crosslinked hydrogels (Figure 20). The PEG-fibrinogen biodegradable hydrogels were covalently conjugated with fluorescent probes for the purpose of monitoring the diffusion of the cyanine-labeled (Cy5.5) fibrinopeptides during gel degradation. In vivo fluorescence analysis of the hydrogels was performed with Xenogen IVIS-200. The applied method provided data regarding release and resorption profiles of degradation products of the hydrogel implants.

Another commercially available imaging system was applied by Lovati et al. for in vivo evaluation of bone deposition on surface-modified titanium implants [125]. In this study, the microporous titanium surface was loaded with bone marrow mesenchymal stem cells (to support implant-bone integration) and hydrogel enriched with strontium (anabolic and anticatabolic properties towards bone). For the purpose of bone deposition monitoring, a fluorescent bisphosphonate OsteoSense^TM^750 was applied. The fluorescence was detected by an eXplore Optix imaging system (ART Advanced Research Technologies). The results showed an increased matrix deposition on stem cells and strontium modified implants.

**Figure 21:**
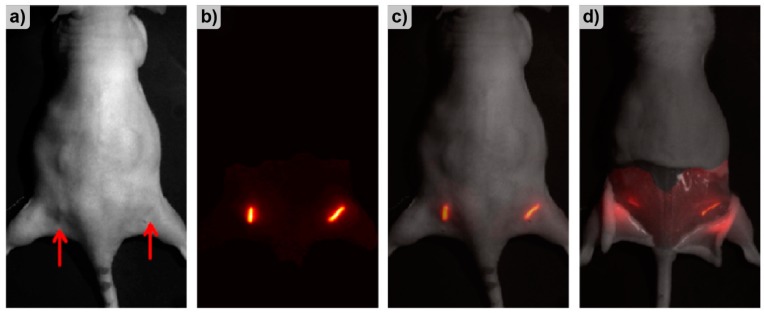
The figure shows (**a**) a white light image of the mouse, (**b**) the normalized fluorescence image, (**c**) an overlay of (**a**) and (**b**) (reference), and (**d**) the residual fluorescence demonstrating continuous diffusion from the spacer over time (image registered after the animal was euthanized and its skin layer and the spacers were removed). Figure adapted with permission from [126].

In a different study, Markovic et al. evaluated a new implantable nanoplatform for drug delivery. The study focused on visualization and quantification of in vivo diffusion of fluorescently doped NPs [126]. To validate the platform, the release of free fluorophores and 30 nm and 200 nm sized NPs conjugated with the same fluorophores as a model drug was studied. The release was studied in agar gel phantoms in vitro and in mice in vivo (Figure 21). PLGA spacers coated with either a free dye, 30 or 200 nm NPs were implanted in the rear flanks of nude mice and, then, imaged daily for 15 days. The fluorescence of the implants was monitored with a custom-designed imaging system (substitute of a commercial system) utilizing NIR fluorescence.

## 5. Conclusions

The complexity of materials and surface functionalities used currently in implant manufacture requires a set of tools capable of providing the modern researcher with the data necessary to thoroughly characterize and profile the research target, with an emphasis placed on the quality of the data, sample preparation, time of the analysis, and cost. Since an ideal, single, and comprehensive method does not exist and seems to be out of reach anywhere in the near future, the scientific community is often turning towards spectroscopic and fluorescent microscopy methods, used separately or in tandem. With their development, scientists have gained access to tools very often capable of assessing properties of samples without the need for special pretreatment or preparation, that can be used both in vitro and in vivo. This possibility is of the utmost importance in modern implantology studies in which the implants surface and its interactions with the surrounding tissue are crucial and responsible for its success.

The complementary applications of spectroscopic and fluorescence microscopy methods are not to be overlooked. Currently we are witnessing progress in developing their combined methodologies, microspectroscopy and spectromicroscopy, capable of spectral measurements directly from microscopic samples or from microscopic images, ensuring the necessary precision and resolution, while leaving the sample intact.

It can safely be said that the position and importance of spectroscopy and fluorescence microscopy among the methods used by the scientific community are not endangered. On the contrary, we should expect further development and expansion of these techniques, including merging with other methods, for example, the combination of atomic force microscopy with IR to yield AFM-IR capable of nanoscale chemical analysis. Additionally, developing alternative methodologies to already existing ones, for example, electrochemical or Raman microscopy label-free biosensing methods should be expected. Further developments will happen with respect to the equipment used and supporting computational methods, offering novel functionalities with improvements in data quality, accuracy, and ease of interpretation.

## Figures and Tables

**Table 1:** Results of the quantitative analysis of the newly formed bone around implants and cortical bone, after four and eight weeks. Table adapted with permission from [52].

Time	Weight %		Ratio
	Ca	P	Ca/P
$4{\;}_{NB}^{weeks}$	15.07 ± 2.83	7.83 ± 1.56	1.93 ± 0.10
$4{\;}_{CB}^{weeks}$	19.77 ± 1.65	10.27 ± 1.05	1.93 ± 0.07
$8_{NB}^{\;weeks}$	17.33 ± 2.39	8.90 ± 0.80	1.94 ± 0.10
$8_{CB}^{\;weeks}$	21.33 ± 1.88	11.03 ± 0.70	1.93 ± 0.05

***NB***, newly bone and ***CB***, cortical bone.

**Table 2:** List of commercially available implants tested. Table reproduced with permission from [69].

Product	Code	Surface Treatment *	Manufacturer
Allfit	ALF	Al_2_O_3_ blasted	Dr. Idhe Dental
Ice	ICE	Smooth machined	3i
IMZ TPS	TPS	Titanium plasma sprayed	Friedrichsfeld
Laser Lok	LLK	Tricalcium phosphate/hydroxyapatite blasted	Biohorizons
PrimaConnex	PRC	Calcium phosphate blasted	Lifecore Biomedical
Ospol	OSP	Calcium-anodized	Ospol
Osseospeed TX	OSS	Titanium oxide blasted/fluoride treated	Astra Tech
Osseotite Full	OTF	Acid-etched (double)	3i
Replace Select	RPS	Calcium phosphate anodized	Nobel Biocare
SLA	SLA	Al_2_O_3_ blasted/acid etched	Institute Straumann
Trilobe	TRB	Al_2_O_3_ blasted	Southern Implants

* According to manufacturers’ information.

**Table 3:** Summary of the fluorescence techniques and their applications in biomaterial studies and implantology.

Fluorescent Technique	Staining Method	Monitored Cells	Monitored Tissues	Principle Of The Study	Ref.
Fluorescence microscopy	Oxytetracycline, alizarin complexion, calcein, xylenol	-	Implant-surrounding fibrous tissue	Evaluation of the osteotome technique	[114]
	Rhodamine-phalloidin, DAPI, fluorochrome-conjugated secondary antibody	rBMSC, HUVEC	-	Evaluation of the osteogenic and pro-angiogenic properties of Zn/Mg coating	[115]
	Bisbenzimide trihydrochloride, phalloidin Atto 488	NIH 3T3	-	Evaluation of the properties and cytocompatibility of silicon carbide ceramics	[116]
	FITC-phalloidin, DAPI, propidium iodide, acridine orange	MC3T3-E1, *S. aureus*, *E. coli*	-	Evaluation of the cytocompatibility and antibacterial properties of ZnO/Ag/PLGA coating	[117]
X-ray fluorescence microscopy	-	-	Implant-surrounding fibrous tissue	Evaluate the level of corrosion of electrode implants	[118]
	-	-	Implant-surrounding bone tissue	Monitoring of the release of cobalt and chromium from Co-Cr alloy implant	[119]
	-	-	Implant-surrounding connective tissue	Monitoring of the release of nickel from nickel-based implants	[120]
Confocal microscopy	Tetracycline hydrochloride, calcein	-	Implant-surrounding bone tissue	Visualization of the process of the new bone formation	[112]
	Phalloidin, DAPI, live/dead assay kit (in vitro), calcein (in vivo)	hMSC	Implant-surrounding bone tissue	Description of the influence of a coating on the new bone formation	[108]
	Immunostaining with goat anti-mouse IgG-FITC, rhodamine-phalloidin, DAPI, Live/Dead Bacterial Viability Kit	MC3T3-E1, *E. coli*	-	Evaluation of cytocompatibility and antibacterial properties of graphene/gelatin-based coating	[121]
Total internal reflection fluorescence microscopy	1,1′-dioctadecyl-3,3,3′,3′-tetramethylindocarbocyanineperchlorate (DiIC18(3))	Bovine aortic endothelial cells	-	Description of dynamics of cell surface adhesion	[122,123]
	1,1′-dioctadecyl-3,3,3′,3′-tetramethylindocarbocyanineperchlorate (DiIC18(3))	Endothelial cells	-	Description of dynamics of cell-implant contact	[122,123]
Imaging systems	Cy5.5 N-hydroxysuccinimide	-	Hydrogel-surrounding soft tissue	Integration of biodegradable hydrogel implants containing VEGF	[124]
	OsteoSense^TM^750	-	Bone tissue accumulated on the implants	Bone deposition on surface-modified titanium implants	[125]
	Cy7.5-NHS ester	-	Implant-surrounding soft tissue	Visualization and quantification of diffusion of fluorescently doped NPs	[126]
